# Stabilization of an elusive tautomer by metal coordination

**DOI:** 10.1107/S2053229621006203

**Published:** 2021-06-22

**Authors:** Emmanuele Parisi, Roberto Centore

**Affiliations:** a Dipartimento di Scienze Chimiche, Università degli Studi di Napoli ’Federico II’, Complesso di Monte S. Angelo, Via Cinthia, 80126 Napoli, Italy

**Keywords:** heterocycle, triazole, tautomerism, elusive tautomer, crystal structure, zinc, copper

## Abstract

The solid-state isolation of the different tautomers of a chemical com­pound can be a challenging problem. In many cases, tautomers with an energy very close to the most stable one cannot be isolated (elusive tautomers). In this article, with reference to the 4-methyl-7-(pyrazin-2-yl)-2*H*-[1,2,4]triazolo[3,2-*c*][1,2,4]triazole ligand, for which the elusive 3*H*-tautomer has an energy only 1.4 kcal mol^−1^ greater than the most stable 2*H* form, we show that metal com­plexation is a suc­cessful and reliable way for stabilizing the elusive tauto­mer. We have pre­pared two com­plexes of the neutral ligand with CuBr_2_ and ZnBr_2_, namely, aqua­bromido­bis­[4-methyl-7-(pyrazin-2-yl)-3*H*-[1,2,4]triazolo[3,2-*c*][1,2,4]triazole]copper(II) bromide trihydrate, [CuBr(C_8_H_7_N_7_)_2_(H_2_O)]Br·3H_2_O, and di­bro­mido­[4-methyl-7-(pyrazin-2-yl)-2*H*-[1,2,4]triazolo[3,2-*c*][1,2,4]triazole][4-methyl-7-(pyrazin-2-yl)-3*H*-[1,2,4]triazolo[3,2-*c*][1,2,4]tri­azole]zinc(II) monohydrate, [ZnBr_2_(C_8_H_7_N_7_)_2_]·H_2_O. The X-ray analysis shows that, in both cases, the elusive 3*H*-tautomer is present. The results of the crystallographic analysis of the two com­plexes reflect the different coordination preferences of Cu^II^ and Zn^II^. The copper(II) com­plex is homotautomeric as it only con­tains the elusive 3*H*-tautomer of the ligand. The com­plex can be described as octa­hedral with tetra­gonal distortion. Two 3*H*-triazolotriazole ligands are bis-chelated in the equatorial plane, while a water mol­ecule and a bromide ion in elongated axial positions com­plete the coordination environment. The zinc(II) com­plex, on the other hand, is heterotautomeric and con­tains two bromide ions and two monodentate ligand mol­ecules, one in the 2*H*-tautomeric form and the other in the 3*H*-tautomeric form, both coordinated to the metal in tetra­hedral geometry. The observation of mixed-tautomer com­plexes is unprecedented for neutral ligands. The analysis of the X-ray mol­ecular structures of the two com­plexes allows the deduction of possible rules for a rational design of mixed-tautomer com­plexes.

## Abstract 2

With reference to a [1,2,4]triazolo[3,2-*c*][1,2,4]triazole ligand, for which the 3*H*-tautomer has an energy only 1.4 kcal mol^−1^ greater than the most stable 2*H*-tautomer, we show that metal com­plexation is a successful and reliable way for stabilizing the higher-energy elusive tautomer.

## Introduction   

Tautomers are structural isomers in ready equilibrium between each other (McNaught & Wilkinson, 1997 ▸). They are intriguing chemical systems that, in a certain sense, can be considered as ‘living mol­ecules’. In fact, because of the equilibrium they undergo, the relative amounts of the different forms in solution can be altered by physical or chemical factors (temperature, solvent, pH, metal ions, *etc*.) through the laws of chemical equilibrium. Tautomers have been central in chemistry since the early work of Berzelius on cyanic and cyanuric acids in 1832, and the discovery of keto–enol tautomerism by Erlenmeyer in 1880 (Taylor *et al.*, 2014 ▸). The issue of tautomerism has been fundamental in many turning points of research in chemistry. For instance, it was fundamental in the discovery of the structure of DNA by Watson & Crick (1953*a* ▸), which relies on the keto-tautomeric forms of purine and pyrimidine bases, and in their seminal hypothesis (Watson & Crick, 1953*b* ▸) that noncanonical tautomeric forms of the bases could be involved in mutagenesis (Goodman, 1995 ▸; Wang *et al.*, 2011 ▸). Now the relevance of tautomers is increasingly recognized in many fields of applied chemistry, including drug design (Martin, 2009 ▸) and materials chemistry (Bussetti *et al.*, 2014 ▸; Horiuchi *et al.*, 2017 ▸). In the realm of coordination chemistry, tautomerism can also be a relevant issue, because some classes of ligands show tautomerism. As an example, keto–enol tautomerism is shown in amino–naphthol derivatives (Deneva *et al.*, 2019 ▸), in di­hydroxy­quinolines (Todorov *et al.*, 2012 ▸) and in aroylhydrazine ligands (Borbone *et al.*, 2004 ▸), while thione–thiol tautomerism is present in di­thio­carbazate ligands (Takjoo & Centore, 2013 ▸). In most of these cases, the common feature is that only one tautomeric form acts as the ligand and is found in the com­plexes.

In the realm of fused-ring heteroaromatic systems that we have studied over the years (Centore *et al.*, 1996 ▸, 1999 ▸; Ambrosanio *et al.*, 1999 ▸), we have found in [1,2,4]triazolo[3,2-*c*][1,2,4]triazole a heterocyclic system with a rich tautomeric behaviour (Centore *et al.*, 2013 ▸, 2015 ▸, 2017 ▸; Fusco *et al.*, 2018 ▸).

**Figure 1:**
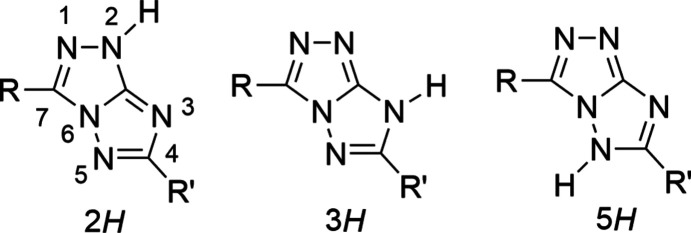
Relevant chemical diagrams for the [1,2,4]triazolo[3,2-*c*][1,2,4]triazole system: the three tautomers of the neutral form (the adopted atom numbering of the heterobicycle is indicated only for the 2*H*-tautomer).

We have found that the relative energy of the three tautomers of the neutral mol­ecule (Fig. 1 ▸) can be significantly modulated by acting on the electronic character of the substituents and on the polarity of the solvent. In all the cases investigated, the energy trend of the tautomers is *E*(2*H*) < *E*(3*H*) << *E*(5*H*); in particular, while the predicted energy of the 5*H*-tautomer is always prohibitive (+10.8 kcal mol^−1^ with respect to the 2*H*-tautomer, in the most favourable case) (Centore *et al.*, 2015 ▸), the calculated energy of the 3*H*-tautomer, in some cases, is greater than for the 2*H*-tautomer by only 1 kcal mol^−1^ or less (Centore *et al.*, 2015 ▸). Despite this, the 3*H*-tautomer should be considered elusive, because it has not yet been observed in the solid state for any of the pure triazolotriazole com­pounds studied so far.

**Figure 2:**
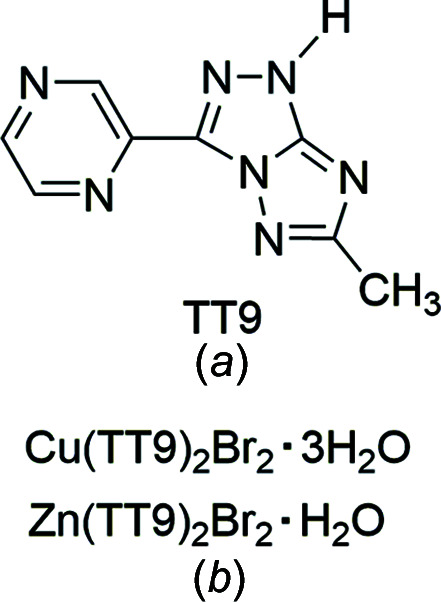
(*a*) Chemical diagram of the triazolotriazole ligand TT9 discussed in this article (the 2*H*-tautomer is shown). (*b*) The chemical formulae of the com­plexes studied in this article.

We have recently described a new versatile nitro­gen-rich tri­azolotriazole ligand, 4-methyl-7-(pyrazin-2-yl)-2*H*,3*H*-[1,2,4]triazolo[3,2-*c*][1,2,4]triazole, henceforth TT9 (Fig. 2 ▸ *a*), whose tautomeric behaviour is further enriched by the possibility of metal coordination (Parisi *et al.*, 2020 ▸).

In particular, while crystallization of the pure neutral ligand yielded the most stable 2*H*-tautomer, as expected, crystallization of neutral TT9 in the presence of Zn^II^ and Cu^II^ salts yielded metal com­plexes of the neutral ligand, with a 1:1 metal-to-ligand ratio, in which the 3*H*-tautomer is present (Parisi *et al.*, 2020 ▸). In order to further confirm metal com­plexation as a way of stabilizing the elusive 3*H*-tautomer, we report, in this article, structural data for the Zn^II^ and Cu^II^ com­plexes of TT9, with a 1:2 metal-to-ligand ratio (Fig. 2 ▸ *b*).

## Experimental   

All reagents were of analytical grade and were used without further purification.

### Synthesis and crystallization   

The synthesis of the TT9 ligand was performed according to the procedure already described by us (Parisi *et al.*, 2020 ▸). Prismatic green crystals of the com­plex Cu(TT9)_2_Br_2_·3H_2_O, henceforth com­plex **1**, were grown in 2 d by slow evaporation of a clear 50:50 (*v*/*v*) water–ethanol solution con­taining a 1:2 molar ratio of CuBr_2_ dihydrate (13.0 mg, 7.63 × 10^−5^ mol) and TT9 (31.0 mg, 1.56 × 10^−4^ mol) at room temperature in a qu­anti­tative yield (53.0 mg). Prismatic brown crystals of the com­plex Zn(TT9)_2_Br_2_·H_2_O, henceforth com­plex **2**, were grown in a week by slow evaporation of a 50:50 (*v*/*v*) water–ethanol solution con­taining a 1:2 molar ratio of ZnBr_2_ (14 mg, 0.1 mmol) and TT9 (40 mg, 0.2 mmol) at room temperature in a 75% yield (48 mg).

### Refinement   

Crystal data, data collection and structure refinement details are summarized in Table 1 ▸. H atoms bonded to C atoms were generated stereochemically and refined by the riding model. After having placed C-bound H atoms, those bonded to O and N atoms, that are essential in the identification of tautomers, were clearly found in difference Fourier maps as the first maxima and, in some cases, their coordinates were refined. For all H atoms, *U*_iso_(H) = 1.2*U*_eq_ of the carrier atom was assumed (1.5 in the case of methyl groups). The structure of com­plex **1**, in the noncentrosymmetric space group *Cc*, was refined as a two-com­ponent inversion twin.

## Results and discussion   

**Figure 3:**
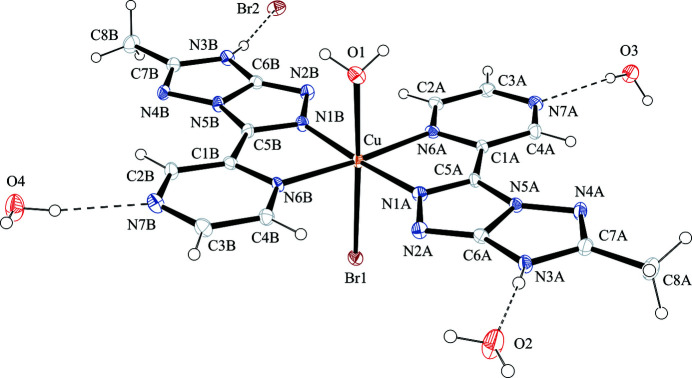
The mol­ecular structure of com­plex **1**, with displacement ellipsoids drawn at the 30% probability level. Selected hydrogen bonds are indicated by dashed lines.

The X-ray mol­ecular structure of com­plex **1** is shown in Fig. 3 ▸. Two 3*H*-tautomeric *s–cis* TT9 neutral ligands are coordinated to copper(II) as bidentate chelates (N1 and N6) in a square-planar arrangement, with the formation of penta­tomic chelate rings. The four metal-to-ligand distances in the equatorial plane show a clear and significant asymmetry. In fact, the bond lengths with N-pyrazinic donors [Cu—N6*A* = 2.117 (5) and Cu—N6*B* = 2.125 (5) Å] are longer than with N-triazole donors [Cu—N1*A* = 1.953 (5) and Cu—N1*B* = 1.951 (5) Å]. This presumably reflects the fact that the pyrazine N atom is a poorer donor. One water mol­ecule and one bromide ion com­plete the coordination environment of Cu^II^, with *trans*-elongated bond lengths of Cu—O = 2.410 (5) and Cu—Br = 2.7466 (12) Å. The coordination geometry can be described as octa­hedral with tetra­gonal distortion. The observed coordination geometry is typical of Cu^II^ and can be related to Jahn–Teller distortions (Cotton *et al.*, 1999 ▸).

The selection of the 3*H*-tautomer, in com­plex **1**, is probably related to the strong preference of Cu^II^ for square-planar coordination with N-donor atoms. The formation of penta­tomic (N1 and N6) chelate rings drives the selection of the *s–cis* conformer and the switching of the proton from N2 to N3. In fact, in the observed mol­ecular structure, there are close intra­molecular con­tacts (weak hydrogen bonds) C2*A*—H2*A*⋯N2*B* and C4*B*—H4*B*⋯N2*A* (Table 2 ▸). Three uncoordinated water mol­ecules and a bromide ion are also present in the crystallographically independent unit. They are involved in strong hydrogen bonds with the N—H donors and N-atom acceptors present on the rim of the coordinated ligands (Table 2 ▸).

**Figure 4:**
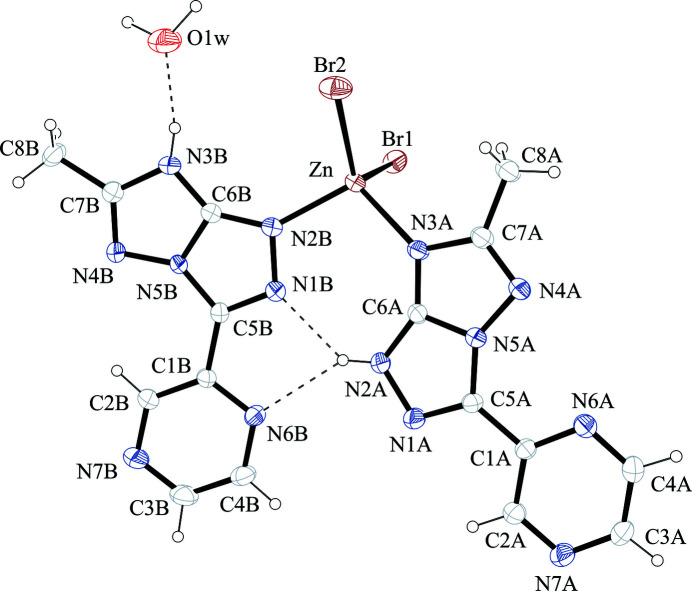
The mol­ecular structure of com­plex **2**, with displacement ellipsoids drawn at the 30% probability level. Selected hydrogen bonds are indicated by dashed lines.

The X-ray mol­ecular structure of com­plex **2**, shown in Fig. 4 ▸, has com­pletely different features. The coordination around Zn^II^ is basically tetra­hedral and is accom­plished through two bromide ions [Zn—Br1 2.4017 (11) and Zn—Br2 2.3581 (9) Å] and two TT9 ligands acting in a monodentate manner [Zn—N3*A* = 2.059 (4) and Zn—N2*B* = 2.018 (4) Å]. The two TT9 ligands are present in different tautomer/conformers. Ligand *A* is 2*H*-tautomeric *s–trans*, whereas ligand *B* is 3*H*-tautomeric *s–cis*. In the com­plex, the two ligands are hydrogen bonded to each other through a strong bifurcated hydrogen bond, N2*A*—H2N*A*⋯N1*B* and N2*A*—H2N*A*⋯N6*B* (Table 3 ▸). Examples of com­plexes in which two different tautomeric forms of the same ligand are coordinated to the same metal centre are rare for anionic ligands (Sutradhar *et al.*, 2016 ▸), and, to the best of our knowledge, not previously documented for neutral ligands.

A deep inspection of the structure of com­plex **2** can suggest some basic points for the rational design of such mixed-tautomeric-ligand com­plexes. The two tautomeric forms should have similar ligand-donor capability and similar energy, in such a way that both are present in solution in similar amounts. They should possess com­plementary functional groups to form stable adducts by secondary inter­actions (*e.g.* hydrogen bonds), with a strong preference for mixed adducts. Finally, the mixed hydrogen-bonded adduct should be featured with a pocket, suitable for the dimensions and presence of donor atoms, to bind a metal ion. The fulfilment of all these issues may account for the rarity of the phenomenon.

**Figure 5:**
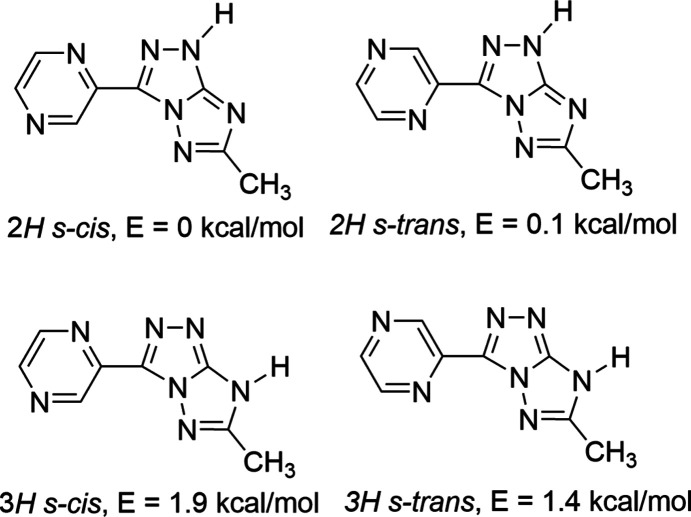
Low-energy tautomers/conformers of TT9. Relative energies are taken from Parisi *et al.* (2020 ▸) and are evaluated in water as the con­tinuum polarizable medium.

**Figure 6:**
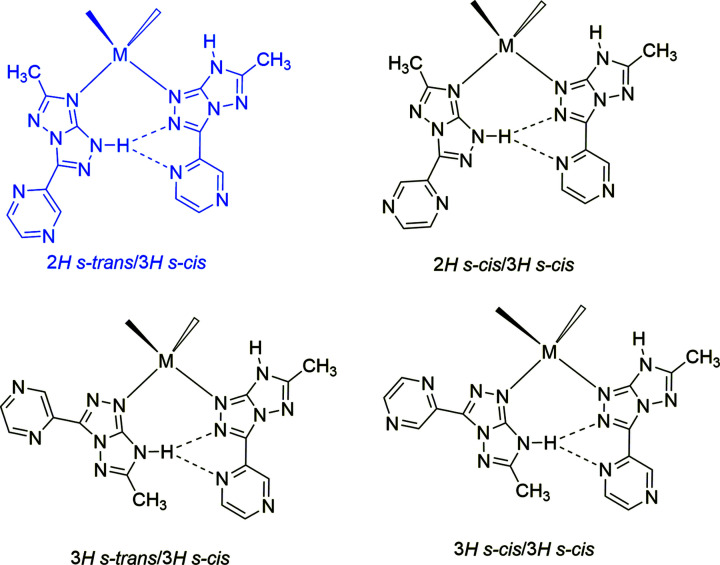
Hydrogen-bonded dimers of neutral TT9. The presence of a metal atom *M* in a tetra­hedral coordination is also outlined. Highlighted in blue is the dimer found in com­plex **2**.

The tautomeric/conformational variability of the TT9 ligand, which possesses four different tautomers/conformers within a narrow energy range Δ*E* < 2 kcal mol^−1^ (Parisi *et al.*, 2020 ▸), makes TT9 a reliable candidate for this target. In fact, we have considered the formation of hydrogen-bonded dimers for the four lowest-energy tautomers/conformers of TT9, shown in Fig. 5 ▸ (Parisi *et al.*, 2020 ▸). Our analysis showed that, of the ten possible combinations, only in four cases can hydrogen-bonded dimers be formed, and they are shown in Fig. 6 ▸. In all the dimers, a strong (bifurcated) hydrogen bond is present between the N—H donor of a tautomer and the two *s–cis* N-acceptor atoms of the other. The hydrogen-bonded dimers also show a pocket with two N-donor atoms that could host a metal ion, for instance, in tetra­hedral coordination geometry. Of the four possible dimers, two are homotautomeric and con­tain the higher-energy 3*H*-tautomer, while the other two are heterotautomeric and con­tain both 2*H*- and 3*H*-tautomers. Evidently, the two latter, 2*H s–trans*/3*H s–cis* and 2*H s–cis*/3*H s–cis*, are energetically more feasible and their energies should be very close, within 0.1 kcal mol^−1^. Thus, our analysis confirms that the formation of mixed-tautomer com­plexes can be expected with TT9.

### Supra­molecular features   

The crystal packing of both com­plexes is basically driven by the formation of a network of strong hydrogen bonds involving N—H donors and N-atom acceptors of the ligand mol­ecules, the bromide ions and the water mol­ecules (see Tables 2 ▸ and 3 ▸).

**Figure 7:**
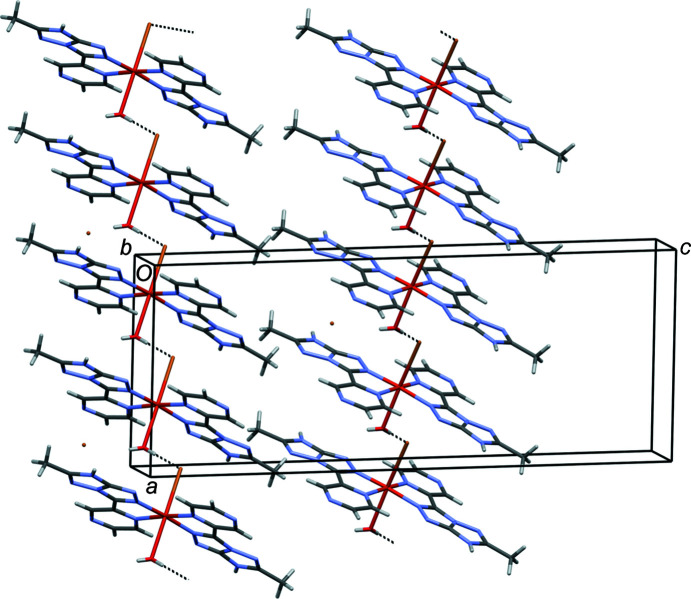
Partial crystal packing of com­plex **1**. Selected hydrogen bonds are indicated by dashed lines.

The equatorial plane of com­plex **1** is neither perpendicular nor parallel to the unique *b* axis. Therefore, in the crystal, mol­ecules with two different orientations are present. The most remarkable supra­molecular architecture is represented by hydrogen-bonded chains running along *a*−*b* and *a*+*b* (Fig. 7 ▸); these directions are equivalent by symmetry in the monoclinic system through the *c*-glide operation perpendicular to the unique *b* axis. The chains are formed by hydrogen bonding between water donors and bromide acceptors axially coordinated to the metal in consecutive com­plex mol­ecules along the chains. Different chains are held together laterally by hydrogen bonds involving noncoordinated water mol­ecules.

**Figure 8:**
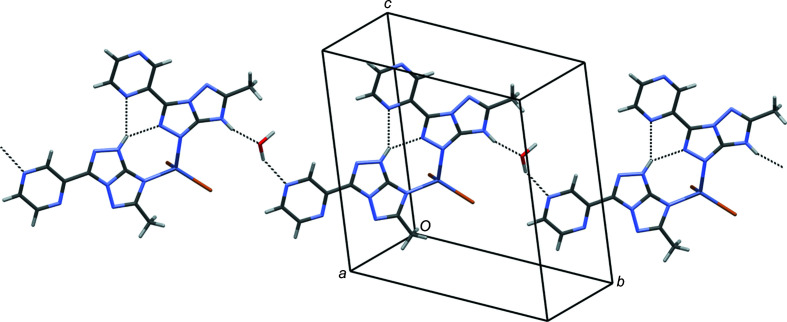
Partial crystal packing of com­plex **2**. Selected hydrogen bonds are indicated by dashed lines.

In com­plex **2**, an intra­molecular bifurcated hydrogen-bonding inter­action is present between the N2*A*—H2N*A* donor group and the N1*B* and N6*B* acceptors of the two coordinated ligands. The most remarkable supra­molecular architecture is represented by nearly planar hydrogen-bonded ribbons of mol­ecules, running along *a*–*b*. The ribbons con­tain 2*H*-tautomeric *s–trans* and 3*H*-tautomeric *s–cis* ligand mol­ecules inter­calated by water mol­ecules (Fig. 8 ▸).

## Conclusions   

Nitro­gen-rich ligand TT9, *i.e.* 4-methyl-7-(pyrazin-2-yl)-2*H*-[1,2,4]triazolo[3,2-*c*][1,2,4]triazole, has a low-energy 3*H*-tauto­mer. While crystallization of pure TT9 from different solvents only affords the most stable 2*H*-tautomer, we have proven that metal coordination allows selection of the elusive 3*H*-tautomer. Depending on the stoichiometry of the com­plex, and on the metal, com­plexes with only the 3*H*-tautomeric ligand (homotautomeric), or com­plexes with mixed-tautomer ligands, *i.e.* both 2*H* and 3*H* (heterotautomeric), can be obtained.

Steering and selectively con­trolling the formation of the different energetically feasible tautomers of a given com­pound, depending on the physico-chemical environment, is of practical and theoretical relevance. In fact, different tautomers generally inter­act differently with the same substrate and show different properties: in an analogy with language, they have different ‘meaning’. After all, chemistry can be considered a language: atoms are its letters and mol­ecules its words. Continuing with this analogy, tautomers, and more generally isomers, would correspond to the anagrams of ordinary language. In this analogy, the heterotautomeric com­plex **2** would correspond to a sentence con­taining two anagrams of the same word, *e.g.* ‘she married an admirer’.

This is uncommon also in ordinary language.

## Supplementary Material

Crystal structure: contains datablock(s) global, 1, 2. DOI: 10.1107/S2053229621006203/jx3062sup1.cif

Structure factors: contains datablock(s) 1. DOI: 10.1107/S2053229621006203/jx30621sup2.hkl

Structure factors: contains datablock(s) 2. DOI: 10.1107/S2053229621006203/jx30622sup3.hkl

CCDC references: 2090148, 2090147

## Figures and Tables

**Table 1:** Experimental details Experiments were carried out with Mo *K*α radiation using a Bruker–Nonius KappaCCD diffractometer. Absorption was corrected for by multi-scan methods (*SADABS*; Bruker, 2001 ▸). H atoms were treated by a mixture of independent and constrained refinement.

	**1**	**2**
Crystal data		
Chemical formula	[CuBr(C_8_H_7_N_7_)_2_(H_2_O)]Br·3H_2_O	[ZnBr_2_(C_8_H_7_N_7_)_2_]·H_2_O
*M*_r_	697.83	645.62
Crystal system, space group	Monoclinic, *C* *c*	Triclinic, *P*\overline{1}
Temperature (K)	173	293
*a*, *b*, *c* (Å)	11.062 (4), 8.369 (3), 27.406 (6)	8.396 (2), 12.305 (3), 12.724 (3)
α, β, γ (°)	90, 92.44 (3), 90	112.53 (2), 107.78 (3), 92.16 (2)
*V* (Å^3^)	2534.9 (14)	1138.1 (5)
*Z*	4	2
μ (mm^−1^)	4.07	4.63
Crystal size (mm)	0.50 × 0.20 × 0.20	0.40 × 0.30 × 0.20

Data collection		
*T*_min_, *T*_max_	0.250, 0.478	0.270, 0.433
No. of measured, independent and observed [*I* > 2σ(*I*)] reflections	8682, 4870, 4563	12306, 5090, 3636
*R*_int_	0.036	0.042
(sin θ/λ)_max_ (Å^−1^)	0.651	0.649

Refinement		
*R*[*F* ^2^ > 2σ(*F* ^2^)], *wR*(*F* ^2^), *S*	0.033, 0.081, 1.04	0.047, 0.136, 1.09
No. of reflections	4870	5090
No. of parameters	367	315
No. of restraints	13	2
Δρ_max_, Δρ_min_ (e Å^−3^)	0.60, −0.86	0.70, −0.74
Absolute structure	Refined as an inversion twin	–
Absolute structure parameter	0.502 (12)	–

Computer programs: *COLLECT* (Nonius, 1999 ▸), *DIRAX*/*LSQ* (Duisenberg *et al.*, 2000 ▸), *EVALCCD* (Duisenberg *et al.*, 2003 ▸), *SIR97* (Altomare *et al.*, 1999 ▸), *SHELXL2016* (Sheldrick, 2015 ▸), *ORTEP-3 for Windows* (Farrugia, 2012 ▸), *Mercury* (Macrae *et al.*, 2020 ▸) and *WinGX* (Farrugia, 2012 ▸).

**Table 2:** Hydrogen-bond geometry (Å, °) for **1**

*D*—H⋯*A*	*D*—H	H⋯*A*	*D*⋯*A*	*D*—H⋯*A*
C2*A*—H2*A*⋯N2*B*	0.95	2.38	3.137 (8)	137
C3*A*—H3*A*⋯Br1^i^	0.95	3.11	4.058 (6)	173
N3*A*—H3N*A*⋯O2	0.86 (3)	1.82 (3)	2.657 (7)	165 (7)
C4*B*—H4*B*⋯N2*A*	0.95	2.52	3.276 (8)	136
C4*B*—H4*B*⋯Br1^ii^	0.95	3.09	3.848 (6)	138
N3*B*—H3N*B*⋯Br2	0.85 (3)	2.34 (3)	3.190 (5)	173 (7)
O1—H1*W*⋯Br1^iii^	0.83 (3)	2.42 (3)	3.229 (5)	166 (6)
O1—H2*W*⋯Br2^ii^	0.83 (3)	2.49 (3)	3.301 (5)	168 (8)
O2—H3*W*⋯Br1^ii^	0.82 (3)	2.43 (3)	3.237 (5)	173 (9)
O2—H4*W*⋯O3^iv^	0.81 (3)	1.89 (4)	2.691 (7)	167 (10)
O3—H5*W*⋯O4^v^	0.79 (3)	2.08 (6)	2.746 (7)	141 (8)
O3—H6*W*⋯N7*A*	0.80 (3)	2.17 (4)	2.931 (7)	159 (9)
O4—H7*W*⋯N7*B*	0.83 (3)	2.12 (3)	2.939 (7)	171 (9)
O4—H8*W*⋯Br2^iv^	0.81 (3)	2.56 (6)	3.272 (6)	147 (8)

Symmetry codes: (i) x, y-1, z; (ii) x+{\script{1\over 2}}, y+{\script{1\over 2}}, z; (iii) x+{\script{1\over 2}}, y-{\script{1\over 2}}, z; (iv) x+{\script{1\over 2}}, y+{\script{3\over 2}}, z; (v) x, -y+1, z+{\script{1\over 2}}.

**Table 3:** Hydrogen-bond geometry (Å, °) for **2**

*D*—H⋯*A*	*D*—H	H⋯*A*	*D*⋯*A*	*D*—H⋯*A*
C2*A*—H2*A*⋯Br1^i^	0.93	3.13	3.995 (6)	155
N2*A*—H2N*A*⋯N1*B*	0.84 (2)	2.09 (4)	2.786 (6)	139 (5)
N2*A*—H2N*A*⋯N6*B*	0.84 (2)	2.39 (4)	3.126 (6)	145 (5)
C2*B*—H2*B*⋯N4*B*	0.93	2.62	3.276 (7)	129
C4*B*—H4*B*⋯Br1^i^	0.93	2.86	3.791 (6)	176
N3*B*—H3N*B*⋯O1*W*	0.85 (2)	1.85 (3)	2.669 (6)	163 (6)
O1*W*—H1*W*⋯Br1^ii^	1.03	2.39	3.375 (6)	162
O1*W*—H2*W*⋯N7*A* ^iii^	0.98	2.03	2.901 (7)	148

Symmetry codes: (i) -x+1, -y, -z+1; (ii) -x, -y+1, -z+1; (iii) x-1, y+1, z.
